# Effects of intraocular lens anterior edge design on anterior capsule morphology changes following femtosecond laser-assisted capsulotomy

**DOI:** 10.1186/s12886-022-02751-x

**Published:** 2022-12-29

**Authors:** Qian Liu, Suhua Zhang, Xiaogang Wang, Jianting Liu, Guohong Zhou, Xiaoyong Yuan

**Affiliations:** 1 Clinical College of Ophthalmology, Tianjin Medical University, Tianjin, China; 2 Tianjin Key Laboratory of Ophthalmology and Visual Science, Tianjin Eye Institute, Tianjin Eye Hospital, Tianjin, China; 3 Cataract department of Shanxi Eye Hospital, Taiyuan, Shanxi Province China

**Keywords:** Anterior capsule, Lens epithelial cells, Cataracts, Femtosecond laser-assisted capsulotomy surgery, Intraocular lens

## Abstract

**Purpose:**

To compare morphological changes in the anterior capsule of two intraocular lenses (IOLs) with different anterior edge designs 6 months after femtosecond laser-assisted capsulotomy surgery (FLACs).

**Methods:**

This study included 168 eyes from168 patients undergoing FLACs. Group A included 74 eyes from 74 patients who had an Acrysof IQ Restor SN6AD3 IOL implantation with a flat anterior edge and Group B included 94 eyes of 94 patients with a TECNIS Multifocal ZMB00 IOL implantation and a "peak-like" anterior edge. All patients were followed up for 6 months. We assessed anterior capsule morphological changes including variation of anterior opening diameters and lens epithelial cell (LEC) proliferation in four directions, variation of anterior opening area, and the level of anterior capsule opacification (ACO).

**Results:**

Variation of anterior opening diameters in 4 directions were significantly lower in Group B (*P* < 0.05). Obvious shrinkage ratio of anterior opening diameters and contraction of anterior opening area (*P* < 0.05) appeared in Group A. LEC proliferation was along the "peak" in Group B, while it spread to the edge of anterior capsule in Group A. ACO grades 6 months after operation in Groups A and B were as follows: grade I in 28.38% and 82.98% of eyes, grade II in 51.35% and 17.02% of eyes, and grade III in 20.27% and 0% of eyes, respectively.

**Conclusions:**

These findings suggest that a "peak-like" IOL anterior edge design played an important role in maintaining the morphology of anterior capsule in the early postoperative stage.

**Supplementary Information:**

The online version contains supplementary material available at 10.1186/s12886-022-02751-x.

## Introduction

Anterior capsulotomy is a crucial procedure in cataract surgery. With the increasing prevalence of the use of multifocal intraocular lenses (IOLs), both the minimization of anterior capsule opacification (ACO) and maintenance of morphological stabilization are critical for improving patient outcomes.

ACO begins within the first month after cataract surgery and typically continues for 6 months. Secondary IOL tilt and decentration can cause a decrease in visual acuity [1]. Even when the procedure is performed smoothly by an experienced surgeon, changes in the anterior capsule are still likely to occur several weeks after surgery [2]. Inconsistency in anterior capsule constriction can result from the imbalance between the centripetal force of anterior capsular fibrosis and the centrifugal force of the zonule [3, 4]. In addition, inconsonant lens epithelial cell (LEC) proliferation produces different centripetal forces in the direction of symmetry, in turn affecting the anterior capsule circularity. Several studies have demonstrated that the design of the IOL, particularly the optic edge, plays an essential role in the process of LEC proliferation [5–7].

To ensure the central position of the IOL, the optic edge of the IOL should be 360° overlapped by the anterior capsule. Femtosecond assistance can enable near-perfect anterior capsulotomy, and it can perform continuous capsulotomy with a level of precision unattainable using manual methods [8], improving the sizing of the capsulotomy by a factor of 12. Previous studies have reported that the use of a femtosecond laser can improve the accuracy of capsulotomy by a factor of 3, as compared with that of manual capsulorhexis. In addition, the tensile strength of the resulting capsule opening is two times greater for femtosecond-assisted procedures than that of the manual procedures [8]. These advantages can maximize IOL performance by ensuring a symmetric, well-centred, and appropriately sized anterior capsulotomy, which is particularly critical for toric, multifocal, and accommodating IOLs. The ability to ensure a perfect capsulotomy for any IOL design is extremely important since capsulotomy construction directly influences the effective lens position [9, 10], which is a significant source of error in IOL power calculation [11]. Researchers have speculated that a sharp optic edge may prevent the proliferation of epithelial cells [12–14].

To the best of our knowledge, no previous studies have examined the effects of IOLs with different anterior edge patterns on the morphology of the anterior capsule following laser-assisted capsulotomy. In this study, we aimed to investigate the anterior capsule morphology changes of the ZMB00 and SN6AD3 IOLs with different optic anterior edge designs 6 months after FLACs.

## Methods

### Patients and criteria

This retrospective observational study included data from 168 patients with age-related cataracts who had undergone FLACs at Shanxi Eye Hospital (China) between March 2017 and June 2018. The research protocols were investigated and authorized by the institutional review board of the Shanxi Eye Hospital. In accordance with the principles of the Declaration of Helsinki, the study was explained to the participants, and written informed consent was provided.

We compared ACO outcomes associated with two types of multifocal IOLs: Acrysof IQ Restor SN6AD3 IOL (Alcon Surgical Inc, Fort Worth, Texas, USA) and TECNIS Multifocal ZMB00 IOL (Abbott Medical Optics Inc, Santa Ana, California, USA). We analysed data for 168 eyes from 168 patients (age range: 34–78 years) exhibiting grade 3 Emery-Little nuclear hardness and significant visual deterioration. Of the 168 patients, 78 were female and 90 were male. The SN6AD3 IOL was used in 74 eyes from 74 patients (Group A) with axial length 23.81 ± 1.03 mm, while the ZMB00 IOL was used in 94 from 94 patients (Group B) with axial length 23.99 ± 1.25 mm. The surgeon remained blinded to the group assignments until implantation of the IOL. All patients were eligible for multifocal IOL implantation based on iTrace standards (Tracey Technologies, Houston, Texas, USA), which specify that alpha and kappa angles must be < 0.5 mm. In addition, astigmatism did not exceed 0.75D in any of the included eyes. Ocular pathologies with risk factors (e.g., retinitis pigmentosa, pseudo-exfoliation syndrome, history of intraocular surgery or inflammation, diabetes mellitus requiring medical control, glaucoma, high myopia) for anterior capsule shrinkage were excluded from this study. The inclusion and exclusion criteria are provided in detail in Table 1.

**Table 1:** Inclusion and exclusion criteria

	Inclusion	Exclusion
Diagnosis	Age-related cataract	Cataract combined with risk factors for anterior capsule shrinkage
Astigmatism	< 0.75D	> 0.75D
Alpha and kappa angles (based on iTrace)	< 0.5 mm	> 0.5 mm
Pupillary diameter after mydriasis	> 6 mm	< 6 mm
FLACs compliance	Can match the body and eye position required by FLACs	Cannot match the body and eye position required by FLACs
Centred and round capsulorhexis	Yes	No

### Examination protocol

Patients were examined 6 months after surgery. LogMAR visual acuity and Best Corrected Visual Acuity (BCVA) were assessed at each visit. Following pupil dilation with 1.0% tropicamide, retro-illumination photographs were obtained using a slit lamp biomicroscope (Topcon CD-3,Japan) to observe morphological variations in the anterior capsule orifice.

Anterior segment optical coherence tomography (OCT) images (AngioVue; Optovue, Inc, Fremont, CA, USA) were obtained in four directions (horizontal,vertical,45°clockwise, and 135°clockwise) across the centre of the IOL in order to detect the proliferation of LECs in each direction. Three OCT measurements were averaged in each direction. The OCT operator was an experienced technician.

The diameter in each direction was measured by importing OCT outcomes into Image J (National Institutes of Health, Bethesda, MD, USA).Variation of diameters in the four directions was evaluated by absolute values of differences between diameter 6 months after operation and 5.2 mm. Shrinkage ratio of anterior diameters were represented by the quantity and percentage of diameter > 5.2 mm. Areas of anterior opening 1 month and 6 months after operation were measured by importing retro-illumination photographs into Image J. Contraction of anterior capsule opening area was compared by absolute values of area differences between 1 and 6 months after operation in two groups.

ACO was graded using the scale proposed by Werner et al. [6]. According to this scale, grade 0 indicates a clear (transparent) anterior capsule; grade I indicates opacification localized at the edge of the capsulotomy; grade II indicates moderate, diffuse opacification, sometimes with areas of capsule folding; grade III indicates intense opacification with areas of capsule folding; and grade IV indicates constriction (phimosis) of the capsulotomy opening (capsulotomy diameter %: 3.5 mm/capsulotomy area %: 9.62 mm^2^).

### IOLs

**Fig. 1:**
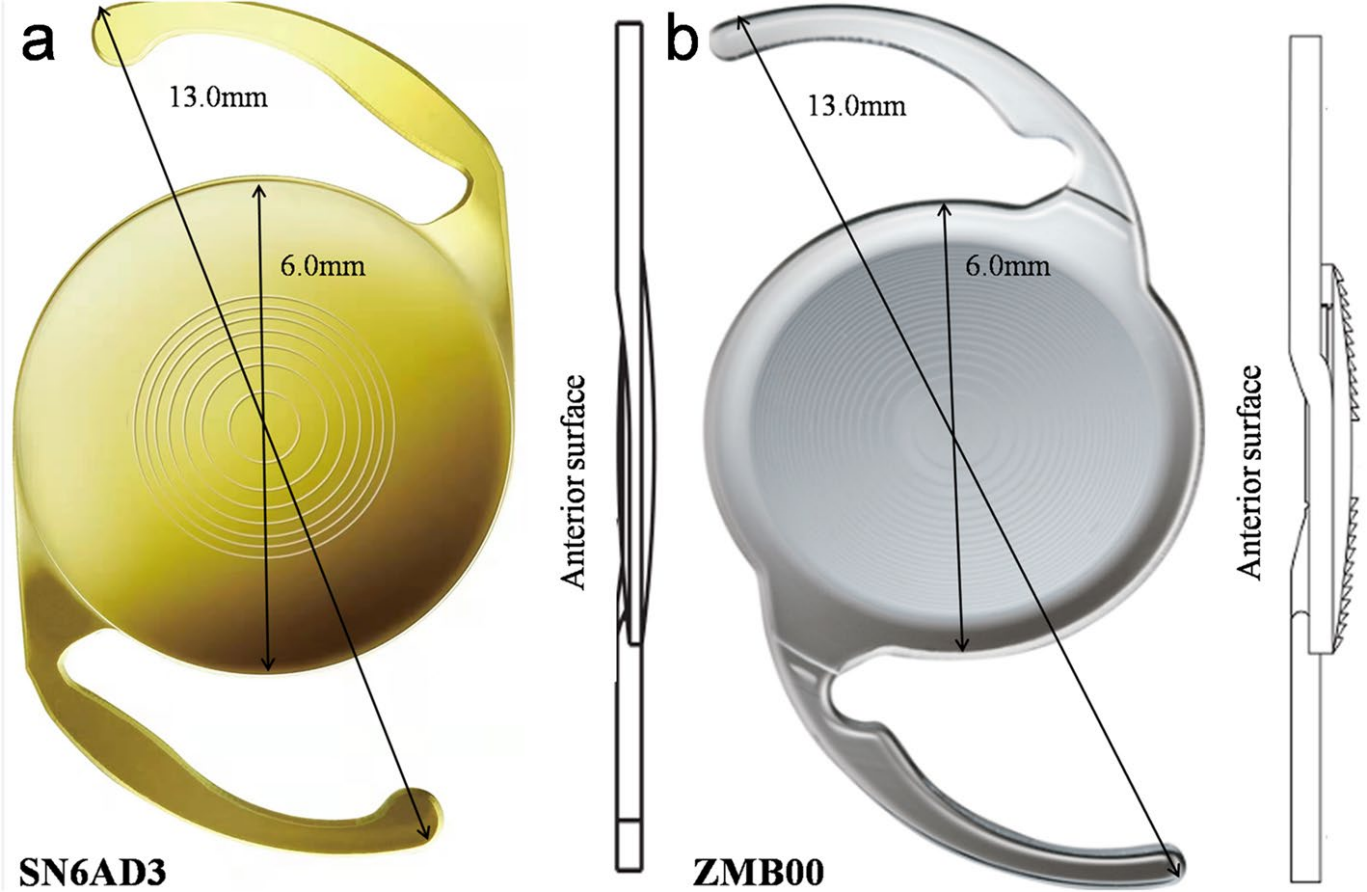
The plane pictures and side structure drawings of SN6AD3 and ZMB00 were showed in a and b respectively. Overall length of the two IOLs were both 13.0 mm and optic diameter were both 6.0 mm from plane view. Haptic angle were both 0° from side view

The two types of IOLs applied in our study were similarly designed as a single piece with C-Loop and square edges. The same overall length and optic diameter were 13 and 6 mm, respectively. Both were made of hydrophobic acrylic material. Dip angle between optic region surface and IOL haptic were both 0°. Plane pictures and side structure drawings of two IOLs are shown in Fig. 1. The Acrysof IQ Restor SN6AD3 IOL (Alcon) exhibits a flat anterior edge, excluding the haptic-optic junction, while the TECNIS Multifocal ZMB00 IOL (AMO) exhibits a continuous 360° "peak-like" anterior edge.

### Surgery

The surgical technique was standardized for each patient. All surgeries were performed by the same experienced surgeon. Following pupil dilation (1 drop of 0.5% tropicamide every 15 min × 4), pupillary diameter was maintained at > 6.0 mm, and a topical anaesthetic (0.5% propantheline HCl) was applied. A femtosecond laser (Alcon-LenSx Lasers Inc, Aliso Viejo, CA, USA) was used to make the primary (2.2 mm) and secondary incisions (1.2 mm) and to perform the capsulotomy, and 7 μJ pulse energy and 6 μm spot separation were used for the capsulotomy. The nucleus was divided using a curved contact lens, following which it was docked and vacuum-suctioned to the cornea. The location of the crystalline lens surface was determined using an OCT imaging system. After scanning in a circular pattern, a 5.20 mm diameter capsulotomy procedure was performed starting at least 100 μm below the anterior capsule and ending at least 100 μm above the capsule. The anterior capsular orifice was entirely isolated in each patient.

In order to observe the LEC proliferation of anterior capsule, anterior polishing was not conducted. To ensure that the optic edge was fully circular and overlapped by the anterior capsule, an infusion needle was placed to make the edge of optical region symmetrical overlapping the anterior capsule prior to making the incision watertight.

### Statistics

Statistical analyses were performed using commercial software (SPSS ver. 13.0; SPSS, Inc., Chicago, IL, USA). The absolute values of the difference between diameter 6 months after operation and 5.2 mm and the absolute values of area differences between 1 and 6 months after operation were not in conformity with normal distribution. The median method of skew distribution was applied for the statistical description in Table 2. The quantity and proportion of diameters greater than 5.2 mm in the four directions of the two IOLs 6 months after operation are displayed in Table 3 and the differences were compared using a chi-square test.

**Table 2:** Differences between diameters in 4 directions and 5.2 mm 6 months after operation and area differences of anterior opening between 1 and 6 months after operation

	**Group A**	**Group B**	**Z**	*P*-value^*^
Horizontal	0.13(0.05,0.28)	0.11(0.04,0.17)	2.083	0.037
Vertical	0.19(0.75,0.34)	0.12(0.05,0.23)	2.334	0.020
45°clockwise	0.18(0.04,0.31)	0.11(0.05,0.17)	2.630	0.009
135°clockwise	0.16(0.08,0.29)	0.09(0.04,0.16)	4.219	< 0.001
Area difference	1.44(0.54,2.19)	0.53(0.24,1.13)	5.248	< 0.001

**Table 3:** Shrinkage ratio of anterior capsule opening in four directions 6 months postoperatively

	**SN6AD3**	**ZMB00**	**X2**	*P*-value^*^
horizontal	34(45.95%)	57(60.64%)	3.600	0.058
vertical	26(35.14%)	54(57.45%)	8.263	0.004
45°clockwise	28(37.84%)	64(68.09%)	15.291	< 0.001
135°clockwise	36(48.65%)	58(61.70%)	2.863	0.091

^*^P-value evaluates the difference of the two intraocular lenses in shrinkage degree of diameters in all four directions

The proportion of patients in different ACO levels 6 months after operation are shown in Table 4. A chi-square test was performed to compare rates and composition ratios. The significance level was set to*p* < 0.05 for all tests.

**Table 4:** Anterior capsule opacification levels of the two groups 6 months after operation

Levels	Group A	Group B	χ^2^	*P*-value^*^
Grade I	21 (28.38%)	78(82.98%)		
Grade II	38 (51.35%)	16(17.02%)		
Grade III	15 (20.27%)	0 ( 0.00%)		
Grade IV	0(0.0%)	0 ( 0.00%)		
Total	74	94	116.954	< 0.001

^*^P-value evaluates statistical differences in percentages of different grades

## Results

**Fig. 2:**
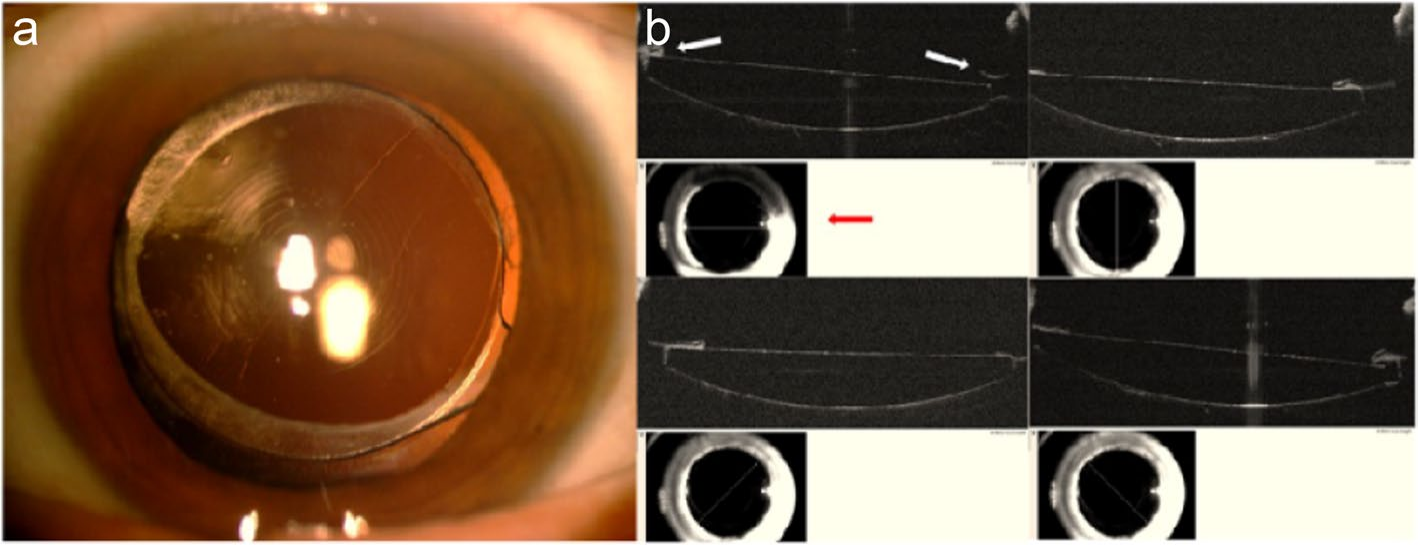
Retro-illumination photograph (**a**) and anterior optical coherence tomography (**b**) in four directions of one eye in Group A six months after surgery. The lens epithelial cell proliferation appears to take place in all regions of interface between the anterior capsule and intraocular lens. Asymmetric proliferation (white arrow) leads to morphological changes of the anterior capsule opening. The scanning direction is depicted using a red arrow

**Fig. 3:**
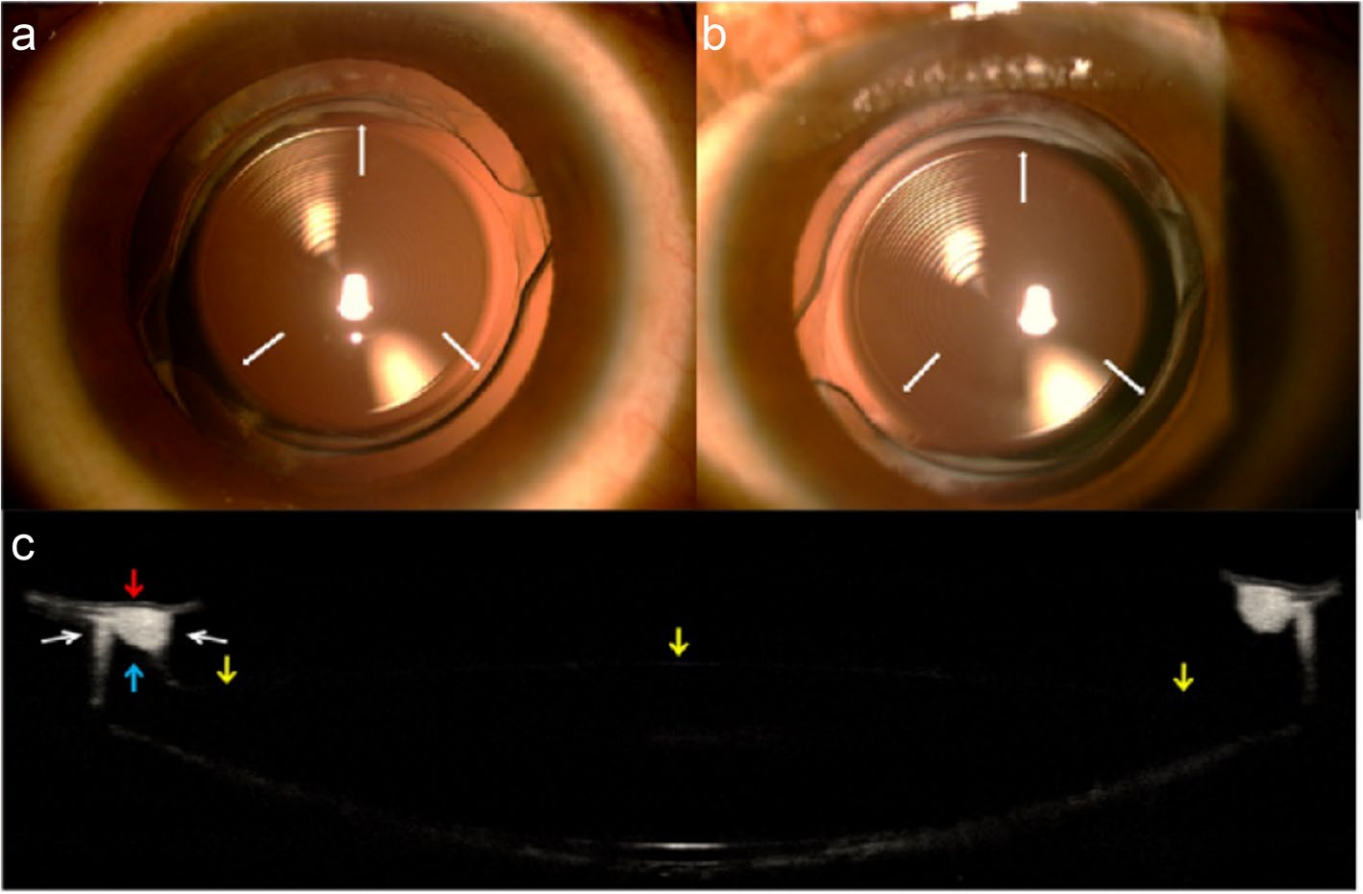
Retro-illumination photograph of the same eye in Group B at one month (**a**) and six months (**b**) after surgery. 3a shows a clear anterior capsule edge and circular anterior capsule opening. 3b displays essentially the same status as that in 3a six months after surgery. Notable, lens epithelial cell proliferation appears limited in the region of the peripheral intraocular lens and capsule. 3c showed anterior optical coherence tomography image of an eye with obvious proliferation in Group B six months after surgery. The edge of the anterior capsule (red arrow) was jacked up by the "peak" (blue arrow) in the meridian bilaterally. The edge cuspappeared clear six months after surgery. Lens epithelial cell proliferation can be seen along both slopes of the "peak" (white arrow). The edge of anterior capsule did not contact the surface of optical region (yellow arrow)

LogMAR visual acuity 6 months after the operation ranged from 0.1 to 0.2 (average: 0.1) in Group A and -0.1 to 0.2 (average: 0) in Group B. BCVA ranged from 0 to 0.2 (average: 0.1) in Group A and -0.2 to 0.2 (average: 0) in Group B. Anterior capsule fibrosis and LEC proliferation were captured by slit lamp biomicroscopy 6 months after the operation. In Group A, LEC proliferation was observed from the edge of the anterior capsule to the peripheral region of the IOL. Such proliferation appeared asymmetric and resulted in different degrees of morphological alteration, as represented in Fig. 2a.In contrast, LEC proliferation in Group B initiated from the "peak" at some distance away from the edge of the anterior capsule opening, and the proliferation was limited to the peripheral regions outside of and around the "peak" as depicted in Fig. 3a. Thus, the edge of the anterior capsule remained clear, and the anterior capsule opening remained circular in shape at the 6-month follow-up in Group B (white arrow in Fig. 3b).

**Fig. 4:**
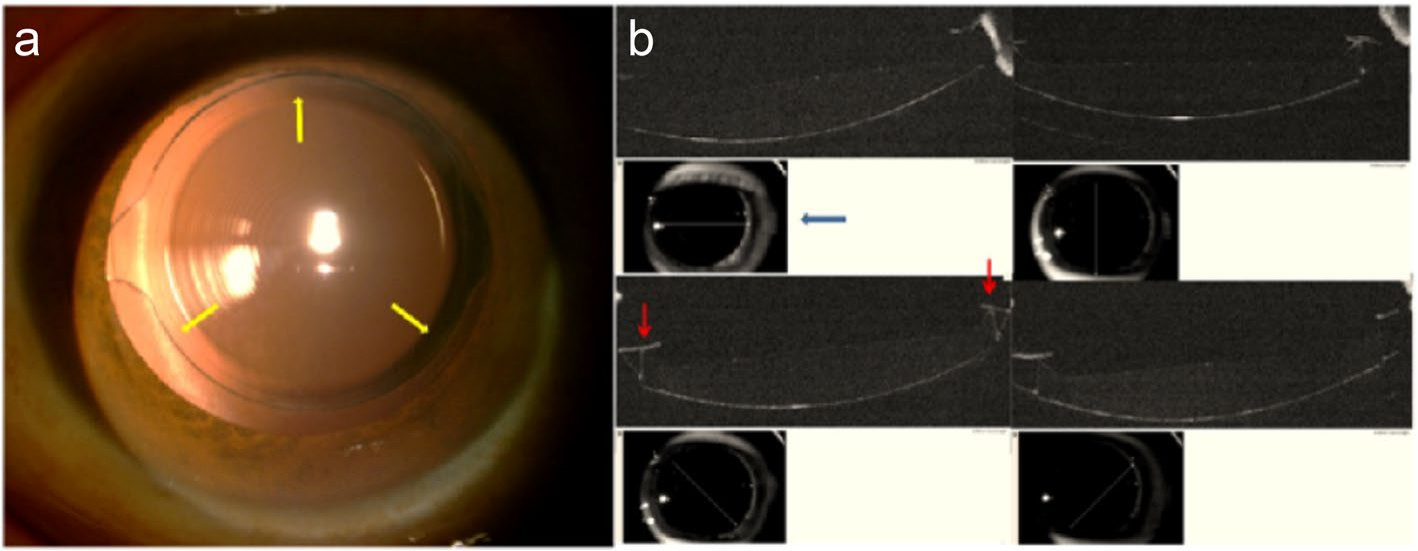
Retro-illumination photograph (**a**) and anterior optical coherence tomography (OCT) in four directions (**b**) of the same eye in Group B at six months after surgery. Anterior capsule orifice maintained round shape (yellow arrow). The contact region of anterior capsule and the intraocular lens maintained two "points" at the "peak" in all four meridians (red arrow). The blue arrow shows the scanning direction of the anterior OCT

Anterior OCT revealed LEC proliferation in the anterior capsule in both groups 6 months after surgery. LEC proliferation was observed in all regions of the interface between the anterior capsule and the IOL. In Group A, such proliferation appeared asymmetric across the meridians of four directions (white arrow in Fig. 2b). However, LEC proliferation appeared to initiate from the top of the "peak" and spread to both slopes of the "peak". The remaining part and the edge of anterior capsule inside the "peak" were discontiguous with the IOL optical region 6 months after surgery in Group B (Fig. 3c). Six months after surgery, the anterior capsule and IOL maintained two points of contact at the "peak" in the meridian of four directions (red arrows in Fig. 4b). LEC proliferation limited around the two points appeared approximately symmetrical in the meridian direction (white arrows in Fig. 3c) and left the edges of anterior capsule clear. Here, LEC proliferation stabilized the anterior capsule orifice in an approximately circular shape (yellow arrows in Fig. 4a).

In accord with the manifestation of Retro-illumination images and anterior OCT, the medians of differences between diameter and 5.2 mm in four directions were lower in Group B than in Group A 6 months after surgery (*P* < 0.05) as shown in Table 2. The quantity and proportion of diameters greater than 5.2 mm were higher in group B in four directions 6 months postoperatively especially in vertical and 45°clockwise directions (*P* < 0.05) as shown in Table 3.

**Fig. 5:**
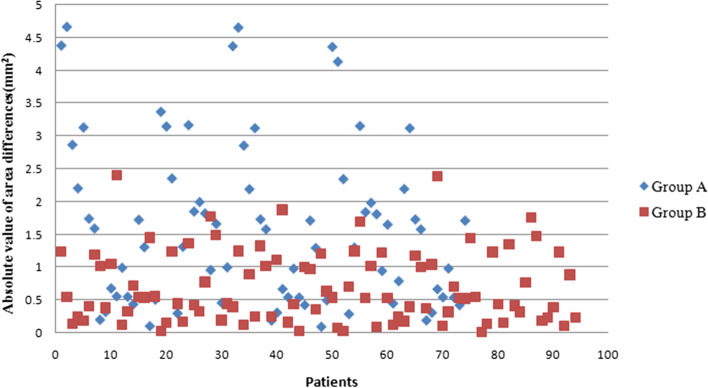
Absolute values of anterior opening area differences between 1 and 6 months after operation in two groups. The distribution of area differences in Group B (red square) was closer to 0 mm2 than that in Group A (blue rhombus)

The distribution of the area difference absolute values in Group B is more concentrated and closer to 0 mm^2^ as shown in Fig. 5. The results are consistent with the statistical outcomes. The median of area differences between 1 and 6 months after operation in Group B is significantly less than that in Group A (*P* < 0.001) as shown in Table 2**,** indicating that the anterior opening areas in Group B were steadier than that in Group A.

According to the scale proposed by Werner et al. [6], ACO reached grade I in 21 of 74 eyes in Group A (28.38%) and 78 of 94 eyes in Group B (82.98%), grade II in 38 eyes in Group A (51.35%) and 16 eyes in Group B (17.02%), and grade III in 15 eyes in Group A (20.27%). No eyes in Group B exhibited grade III ACO 6 months after surgery, and no eyes in either group exhibited grade IV ACO. These findings are summarized in Table 4. The difference in distribution of the four ACO levels of the two groups was statistically significant (*p* < 0.001). LEC proliferation occurred in the anterior capsule in both groups, none of the 168 eyes exhibited capsule folding.

## Discussion

The contraction of the anterior capsule opening represents a potential complication of cataract surgery, and it can lead to vision decrease and IOL decentration [15]. In this study, we evaluated the effects of two hydrophobic IOLs with different anterior edge designs on ACO and morphological stability of the anterior capsule following FLACs. Our findings suggest that a "peak-like" anterior edge design is more effective than a flat design in preventing LEC spread towards the edge of anterior capsule opening and maintaining the morphology of the anterior capsule opening in the early stage after operation.

The objective of this study was to compare the effects of different IOL anterior designs on the capsule opening after FLACs. In order to avoid other influences on the morphology of anterior capsule opening, diseases with pathological factors of suspension ligament relaxation and proliferation of IECs were excluded. E.g., retinitis pigmentosa, pseudo-exfoliation syndrome, history of intraocular surgery or inflammation, high myopia, glaucoma, and diabetes mellitus requiring medical control [16]. All enrolled patients had age-related cataract alone.

When in contact with the IOL, LECs produce cytokines that can induce fibrosis in the anterior capsule [17]. Corydon et al. [18] found that obvious shrinkage of capsulorhexis appeared after surgery with hydrophobic acrylic IOL. Kahraman et al. [19] deduced that Acrysof IOLs showed a tendency towards more ACO than Tecnis IOLs did. However, the result was not statistically significant. Tognetto et al. [20] regarded the biocompatibility of the IOL to be a crucial factor in the process of ACO and ruled out the possibility of the IOL design effects on the anterior capsule behaviour. However, blocking LEC proliferation, migration, and differentiation can prevent contraction of the anterior capsule. Postoperative fibrosis of residual anterior LECs can occur if such cells come in contact with the anterior surface of the IOL [21].

The two hydrophobic IOLs in our study are one-piece designed with equal total length (13.0 mm) and optical diameter (6.0 mm) and the haptic angle were both 0° as shown from the plane and side views in Fig. 1. However, the haptic of ZCB00 is designed with an offset from optic as shown from the side view in Fig. 1. Besides, there are other detail differences in haptic design (length, thickness, surface area, and volume) and optic–haptic junction [22]. Sacu et al. indicated that haptic design of hydrophobic IOLs have no impact on the incidence of ACO or anterior capsular contraction [23]. Meanwhile, early research reported that the optic and loop design of one-piece acrylate IOLs is unrelated to contraction of anterior capsule opening [24].

The relation between contraction of capsule opening and the design of IOL has been reported in several studies. Corydon et al. [18] found that there was no significant difference in contraction of capsule opening between a hydrophobic acrylic IOL (AcrySof MA60AC) with a sharp anterior edge and a hydrophobic acrylic IOL (Sensar AR40e) with a modified anterior edge. Sacu et al. [7] described that the silicone IOL (ClariFlex OptiEdge) with a sharp edge resulted in a more significant contraction than the silicone IOL (PhacoFlex SI-40) with a round edge and two other acrylic IOLs (Sensar OptiEdge AR40e, AR40). The influence of anterior surface design such as "peak" on anterior capsule morphology changes has not been reported. In contrast to previous studies, a continuous capsulotomy of high-level precision could be produced through FLACs in this study, and the interference factors induced by manual capsulorhexis were excluded [8].

The anterior edge of the IOL used in Group B featured a fully circular "peak", which helped to maintain the circular shape of the anterior capsule. In the present study, fibrosis was initiated from the point of contact between the top of the "peak" and the anterior capsule, following which it spread along both slopes of the peak. The edge of the anterior capsule was kept discontiguous with the surface of optical region with no fibrillation 6 months after surgery (Fig. 3c). Thus, the position of the IOL remained fixed, and the circular shape of the anterior capsule opening was maintained. Retro-illumination images further revealed that the edge of the anterior capsule remained clear at the 6-month follow-up, with some distance between the edge of the anterior capsule and the region of proliferation (Fig. 3b). A point-to-point localized contact between the anterior capsule and the IOL was also observed in view of section along the meridian direction (Fig. 4b). This point-to-point pattern ensured a more symmetrical stretch force, even in cases of relatively asymmetrical overlap.

In contrast, the IOL used in Group A exhibited a flat anterior optic periphery. In this group, the anterior capsule adhesions were asymmetrical along the meridian. Anterior OCT revealed different patterns of fibrosis in the direction of symmetry, resulting in different contraction forces along the meridian and subsequent changes in the shape of the anterior capsule 6 months after surgery (Fig. 2). In Group B, LEC fibrosis did not occur along the optical region of the IOL, instead, it occurred along both slopes of the "peak". Consequently, the area between the edge of anterior capsule and the "peak" remained relatively clear (Fig. 3b). The "peak" changed the proliferative pattern of anterior capsule LEC. The fibrosis was limited to the area near the "peak" and in the peripheral capsule and had no obvious effect on the circular structure of anterior opening (Fig. 3c).

To evaluate the stability of the circular shape of the anterior capsule opening, we compared variations in meridian diameter in four directions (every 45°) between the two IOLs. Variation was represented as the absolute value of the difference between meridian diameter and 5.2 mm. The medians were significantly lower along four directions in Group B than in Group A as shown in Table 2. The result illustrated the variations of meridian diameters in four directions compared with 5.2 mm was less in Group B than in Group A and consistent with findings obtained using OCT and retro-illumination images.

In contrast with the report by Kahraman et al. [19], in present study, the variation in four directions of capsule opening in Group B with Tecnis IOL was significantly less than that in Group A with Acrysof IOL. The quantity and percentage of diameters greater than 5.2 mm were larger in 4 directions in Group B than that in Group A, which represent the contraction of diameters in 4 directions was significantly lower in Group B than in Group A in 4 directions and statistically significant in vertical and 45° clockwise directions as shown in Table 3 (*P* < 0.05). The result was consistent with OCT manifestation as showed in Fig. 3c, the edge of the anterior capsule in Group B remained clear 6 months after surgery, and "peak" design decreased the contribution of LEC proliferation on anterior capsule morphology changes. Similarly, to evaluate the stability of anterior opening, area differences between 1 and 6 months postoperatively were compared. The distribution of area differences in Group B were closer to 0 mm^2^ than Group A as showed in Fig. 5. Also, the medians of area difference in Group B were smaller than Group A in 4 directions (*P* < 0.05) as shown in Table 2. Therefore, the area of anterior opening in Group B was steadier.

These above results suggest that the shape of the anterior capsule was more uniform and stable in Group B than in Group A, which was consistent with the result of Werner scale grades. Due to the "peak-like" design in Group B, the rate of grade I was high at 82.98% in Group B compared to 28.38% in Group A in Table 4. Fibrosis originating from the point contacted with anterior capsule was limited along two slopes of the peak (Fig. 3c). Opacification localized on the anterior capsule appeared mild in the early postoperative stage as shown in Fig. 4a. Patients with moderate and intense opacification were less in Group B than in Group A that led to a significant reduction in the proportion of other grades.

In conclusion, our results suggest that the "peak-like" design of the fully circular anterior surface margin acted as a frame for extending the residual LECs in the early postoperative stage. LEC proliferation pattern was changed by "peak" design to maintain the anterior capsule opening as a more stable circle shape than that with flat design. Advantages of this design could benefit patients with a risk of anterior capsule contraction. However, too large, too small, and eccentric capsulorrhexis will disable the "peak-like" design. ICEs would proliferate irregularly in meridian directions and break the force balance of capsular contraction. FLACs is recommended as a key supplement for precise capsulorhexis. We did not examine the relationship between haptic position, haptic angle of contact, and shrinkage ratio of the anterior capsule in different directions in two groups. Moreover, it is not clear whether anterior polishing and the diameter of anterior capsule opening in FLACs are important factors that exert great influence on anterior capsule morphology. More in-depth studies with longer follow-up periods and greater sample size are required to appropriately verify this conclusion. Additionally, the relationship between morphological changes of the anterior capsule and the refractive stability after IOL implantation is an important part of our future research.

## Supplementary Information

**Additional file 1.**

## Data Availability

The datasets used and analysed during the current study are available from the corresponding author on reasonable request.
